# Anaesthesia-induced atelectasis in a rat model (*Rattus norvegicus*)

**DOI:** 10.3389/fvets.2025.1703110

**Published:** 2026-01-09

**Authors:** A. Piskovská, K. Kraszewska, M. Gajewski, M. Skoric, V. Jekl

**Affiliations:** 1 Jekl & Hauptman Veterinary Clinic, Brno, Czechia; 2 Department of Pharmacology and Pharmacy, Faculty of Veterinary Medicine, VETUNI Brno, Brno, Czechia; 3 Vetcardia Veterinary Clinic, Warsaw, Poland; 4 Department of Pathology and Parasitology, Faculty of Veterinary Medicine, VETUNI Brno, Brno, Czechia

**Keywords:** anaesthesia, atelectasis, isoflurane, lung, rat, ultrasound

## Abstract

**Introduction:**

Anaesthesia-induced atelectasis has been observed in many animal species, developing during intravenous or inhalational anaesthesia, with or without mechanical ventilation. Ultrasonography of the lungs is a rapid and non-invasive method that has been shown to be effective in detecting atelectasis. This article aimed to describe anaesthesia-induced atelectasis diagnosed through lung ultrasound in a rat model under isoflurane anaesthesia.

**Methods:**

A total of 12 clinically healthy, 6-month-old rats (*Rattus norvegicus*, Wistar rat; body weight 509.4 ± 109.6 g; mean ±SOD) were used in this study. Lung ultrasound was performed at three time points: (i) 5 min before anaesthesia; (ii) during induction of anaesthesia, when the animal was already wearing the facial mask—immediately after the loss of the righting reflex; and (iii) 2 min after the loss of the righting reflex. Moreover, 5-s video loops were recorded at each time point. At the end of the experiment, the animals were euthanised and subjected to histopathological examination. A modified version of the Oricco’s lung ultrasound score (LUSS) was adapted for use in rats. The video loops were anonymised by the operator and re-scored by two independent scorers to obtain the interobserver agreement score.

**Results:**

The LUSS of all conscious animals showed no pathological changes in the lungs. The adapted LUSS proved to be easy to understand and was consistently applied by all observers. The analysis showed a moderate level of agreement between experts in the assessment of recordings obtained before anaesthesia (0.40 < Kendall’s tau < 0.59) and a very high level of agreement in the assessment of recordings obtained during the induction of anaesthesia and 2 min after the onset of anaesthesia. Additionally, there was a strong consensus regarding the overall assessment of recordings obtained before, during, and after anaesthesia (0.80 < Kendall’s tau < 1.00). Histopathological examination revealed no abnormal findings in any of the animals.

**Conclusion:**

In conclusion, anaesthesia-induced atelectasis can lead to marked ultrasonographic changes in gravity-dependent areas in a rat model anaesthetised with isoflurane. The modified version of Oricco’s LUSS showed significant interobserver agreement, indicating its potential reliability for thoracic ultrasound examinations in laboratory and pet rats.

## Introduction

Lung atelectasis refers to the partial or complete collapse of the lung tissue, leading to a loss of aeration in otherwise structurally normal lung parenchyma (1). The most clinically evident pathophysiological effects of pulmonary atelectasis are hypoxaemia and reduced lung compliance, which increase postoperative morbidity (2). The pathophysiological processes underlying atelectasis develop through three main mechanisms: passive, adhesive, and resorptive (3). Anaesthesia-related atelectasis is complex and caused by a combination of these mechanisms. It can develop during intravenous or inhalational anaesthesia, with or without the use of mechanical ventilation (4).

Anaesthesia-induced atelectasis has been documented in various animal species, such as dogs (5–7), cats (8), sheep (9), horses (10–12), pigs (13), and rabbits (14). In human medicine, anaesthesia-induced atelectasis can occur in patients of all ages, from neonates to older adults (15, 16). Neonates and younger children are at higher risk for anaesthesia-induced atelectasis, due to their increased metabolic demands and lower functional residual capacity, which makes them as more susceptible to hypoxaemia (17, 61).

The diagnosis of atelectasis is based on computed tomography (CT), radiography, and lung ultrasonography. Other methods such as volumetric capnography, electrical impedance tomography, and blood gas analysis can be used to monitor respiratory function (18). Atelectasis can be detected on a CT scan in nearly 90% of patients who are anaesthetised in human medicine (19). Anaesthesia and sedation can cause atelectasis, which may mimic lung pathologies on CT scans (20). An experimental study on dogs found that CT images taken during sedation showed a smaller effect compared to those affected by atelectasis caused by general anaesthesia (20, 21). Opacification related to a lung segment or lobe is a typical finding in atelectasis. Mediastinal shift and compensatory hyperinflation of the expanded lung are classic findings observed in humans and dogs (5, 22). Lung ultrasonography is a fast and non-invasive method that has been shown to be effective in the detection of atelectasis (23–28, 62). As CT is the gold standard for the diagnosis of atelectasis, the main advantage of lung ultrasonography is its ability to be performed at the bedside.

A search of the ScienceDirect and PubMed databases using the terms “rat” AND “anaesthesia” AND “atelectasis” on 28 July 2025, along with a review of current textbooks, revealed only cases of anaesthesia-induced atelectasis described in rat models. These cases are usually associated with the recruitment manoeuvre or a biochemical study of the surfactant (29–33). None of these studies used lung ultrasound for diagnosis, and no monitoring or assessment of the atelectatic lung region was performed.

The aims of this study were (i) to investigate isoflurane-induced atelectasis in laboratory rats using lung ultrasound, (ii) to develop a rat-specific modification of the lung ultrasound score (LUSS), and (iii) to validate the ultrasound findings and the adapted scoring system by analysing interobserver agreement.

## Materials and methods

### Animals

A total of 12 clinically healthy, 6-month-old rats (*Rattus norvegicus*, Wistar rat), 6 male and 6 female rats, were used in the study. The weight of the animals ranged from 357 g to 651 g (509.4 ± 109.6 g; mean ±SOD). The animals were housed individually in polycarbonate ventilated cages in an animal care facility under controlled conditions (12 h daylight/darkness, temperature 18–21 °C, and humidity 42–52%). All rats were fed a pelleted diet ad libitum, and water was offered in drinking bottles. The animals were housed and treated in accordance with the Branch Commission for Animal Welfare of the Ministry of Agriculture of the Czech Republic (45450/2019-MZE-18134). The health status of the animals was assessed on the day of arrival and again 2 days before the procedure through clinical examination, blood analysis, coprology (faecal flotation), and urinalysis.

### Data collection/study protocol

#### Lung ultrasound

Lung ultrasound examination was performed on conscious (non-sedated) animals according to the RATTUS protocol (34) using a linear multifrequency probe (10–16 MHz, Vinno, R700 VET, China, with MI 0.5–1.2, TIS 0.1–0.4). The examiner carefully restrained the conscious rat in sternal recumbency with one hand, while the other hand held the probe. In the anaesthetised animals in sternal recumbency, due to the size of the animals, the probe was placed transversely to the ribs to assess the parasternal-axillary region, where gravity-induced atelectasis was expected. Manipulation with the animals was minimal to stay in the sternal recumbency and persist in the standard position without changing the gravity-dependent site. The linear probe measured 5 cm to 1 cm. Lung ultrasound was performed by a single operator (AP) at three time points: (i) 5 min prior to anaesthesia; (ii) during induction when the animal was already on the facial mask—immediately after the loss of the righting reflex; and (iii) 2 min after the loss of the righting reflex. Moreover, 5-s video loops were recorded at each time point. A single linear probe was used, and the right side was examined first. Loops from the left side were recorded with a 5-s delay. The parameters persistence and detail were lowered, and the gain was set to the distal field of the ultrasound image to obtain a “grainy” image. Harmonisation parameters were turned off. The focal zone was set at the pleural level. With these settings, the lung probe mechanical index (MI) was 1.2 and the thermal index for soft tissue (TIS) was 0.4. The ultrasound frequency was 10 MHz, with a depth of 3.5 cm. Acoustic power was set to 100%, frequency to 29.5 Hz, and dynamic range to 90 dB. The thoracic area was scanned in the axillary region, from the ventral border (sternal and pectoral muscles), via the cranial border (brachial muscles), to the caudal border (abdominal curtain sign).

#### Lung ultrasound scoring system

**Figure 1:**
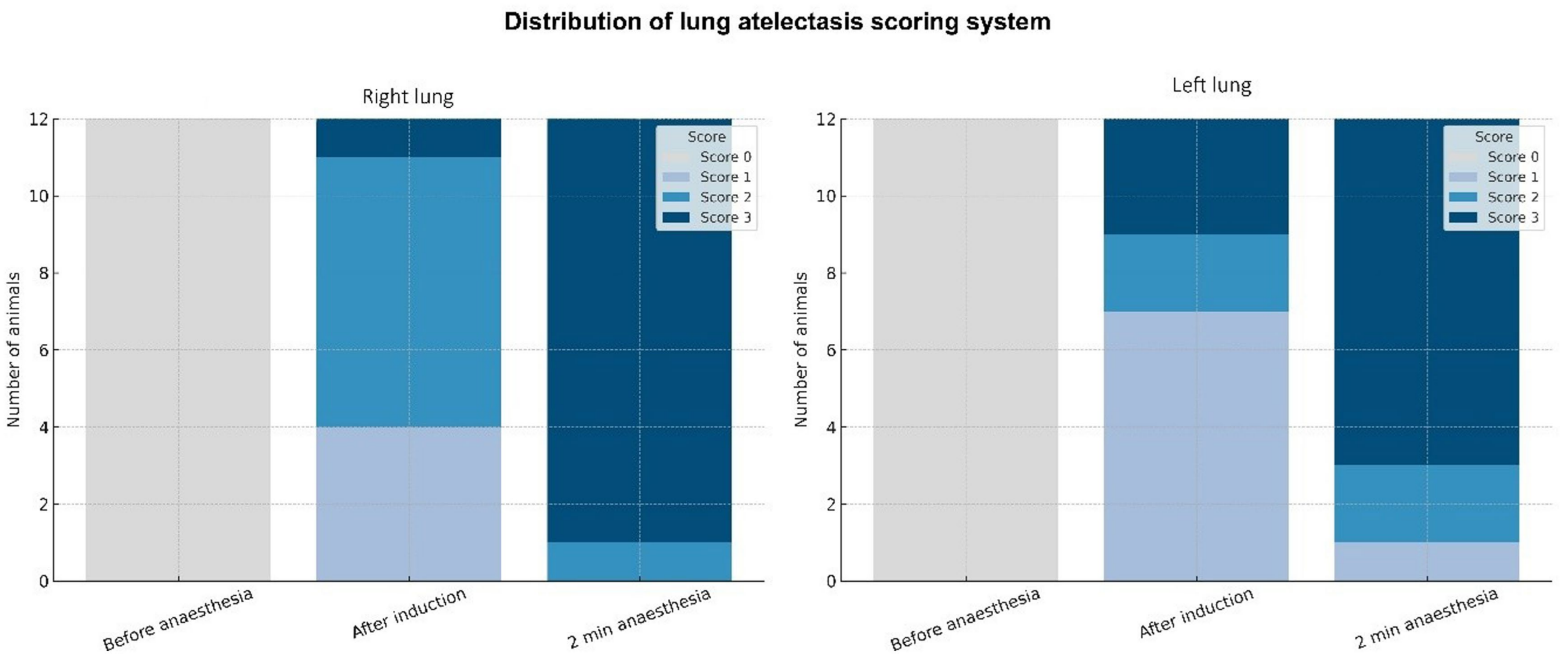
Modified version of Oricco’s lung ultrasound score (LUSS): A score of 0 indicated normal lung aeration, characterised by a continuous pleural line with visible A-lines. A score of 1 represented mild loss of aeration, with vertical artefacts occupying less than 25% of the pleural surface. A score of 2 represented moderate loss of aeration, with vertical artefacts involving 25–50% of the pleural surface. A score of 3 corresponded to severe loss of aeration, characterised by vertical artefacts occupying more than 50% of the pleural surface or the presence of airless subpleural lung tissue (ASLT).

For this study, a modified version of the LUSS (Figure 1) was adapted based on the scoring system developed by Oricco et al. (35). In this modified LUSS, a score of 0 indicated normal lung aeration, characterised by a continuous pleural line with visible A-lines. A score of 1 represented mild loss of aeration, defined as vertical artefacts occupying less than 25% of the pleural surface. A score of 2 represented moderate loss of aeration, with vertical artefacts involving 25–50% of the pleural surface. A score of 3 corresponded to severe loss of aeration, characterised by vertical artefacts occupying more than 50% of the pleural surface or the presence of airless subpleural lung tissue (ASLT).

### Anaesthesia

Anaesthesia was induced with isoflurane in the induction chamber (5%, Isoflutek 1,000 mg/g, Laboratorios Karizoo, S.A., Spain). After the animal showed signs of sedation (discoordination), anaesthesia was maintained via a face mask (2% isoflurane, oxygen flow rate 2 L/min, Eickemeyer anaesthesia masks, size 1, OD: 44 mm, ID: 22 mm, depth: 24 mm), with FiO_2_ of 95% supplied by an oxygen generator. The animal was monitored by visual inspection and auscultation.

### Histopathological examination

The animals were humanely euthanized immediately after the ultrasound examination by intravenous administration of 0.5 mL T61 (MSD, Spain). After post-mortem examination, both lungs were placed in a container with formalin for 3 days and submitted for histopathological analysis. All samples were blinded, and random numbers were generated using a random number generator.^1^ The samples were fixed in buffered 10% neutral formalin, dehydrated, embedded in paraffin wax, sectioned on a microtome at a thickness of 4 μm, and stained with hematoxylin and eosin (H&E). A total of 10 fields were viewed for assessment. Tissue sections were examined using a light microscope (Olympus BX51, Japan).

### Statistical methods

The operator evaluated the video loops for the primary statistical analysis after they were anonymised and randomised (AP; 3 years of experience in lung sonography, trained by KK and human lung ultrasound experts). The loops were then forwarded to two independent evaluators (KK and MG; experienced clinicians with >10 years of echocardiographic practice and 5 years of lung ultrasound experience, including advanced training by human LUS experts). The evaluators assessed and scored the recordings using the modified LUSS. Randomisation was performed using an online random sequence generator (see text footnote 1). After the submission of the ratings, the video loops were decoded according to the reference key and the original data sets were compiled for statistical analysis.

The group size was established based on the hypothesis that atelectasis would be observed in at least 25% of animals. With 11 animals and a 95% CI, the chance of observing at least one case was 95.8%. For the exact analysis, a total of 12 rats were used.

Statistical analyses were performed using IBM SPSS Statistics, version 30 (IBM Corp., Armonk, NY, USA). Descriptive analyses and graphical visualisations (stacked bar charts and boxplots) were performed using Python (version 3.11) and the Matplotlib and Pandas libraries. For the statistical description of the ordinal frequency data, the results were presented as medians with interquartile ranges (IQRs) and percentage distributions. For illustrative purposes, the mean value and standard deviation (SD) were also calculated, whereby the values were treated as quasi-interval data. Due to the ordinal nature of the scores, interobserver agreement was assessed. Kendall’s tau concordance coefficients and 95% confidence intervals were calculated. A *p*-value of <0.05 was considered statistically significant.

## Results

### Lung ultrasound operator evaluation

**Figure 2:**
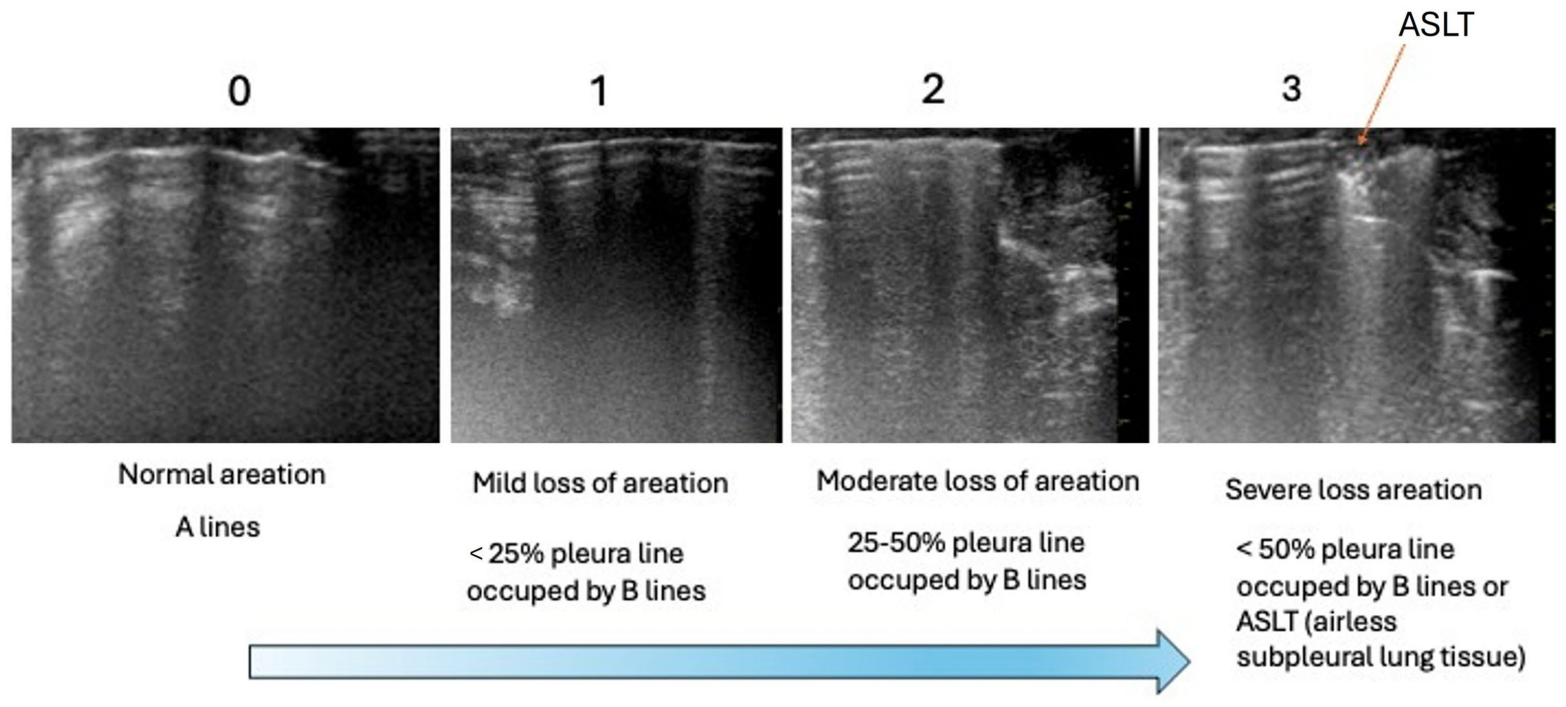
Distribution of the atelectasis score for the right and left lungs of the rats at three time points: (i) 5 min before anesthesia; (ii) during induction, when the animal was already on the facial mask—immediately after the loss of the righting reflex; and (iii) 2 min after the loss of the righting reflex. The results were obtained by a single investigator (AP) who examined the rats before and during anesthesia.

The LUSS of all conscious animals revealed no pathological changes in the lung parenchyma, pleural cavity, or signs of heart disease. In the right lung area, all animals had an LUSS of 0 (median 0, IQR 0–0) before anaesthesia. After induction, the median score increased to 2 (IQR 1–2), with 58.3% of the rats showing a score of 2, and the mean ± SD was 1.75 ± 0.62. After 2 min of anaesthesia, 91.7% of the animals achieved a score of 3, with a median of 3 (IQR 3–3) and a mean ± SD of 2.92 ± 0.29. For the left lung field, all animals had a score of 0 (median 0, IQR 0–0) before anaesthesia. After induction, the median score increased to 1 (IQR 1–2.5), with 58.3% of the rats scoring 1 and a mean ± SD of 1.67 ± 0.89. After 2 min of anaesthesia, 75.0% of the rats achieved a score of 3, with a median of 3 (IQR 2.5–3) and a mean ± SD of 2.67 ± 0.65 (Figure 2).

### Interobserver agreement

The adapted lung ultrasound scoring system for the rat model proved to be easy to understand and was consistently applied by all observers. The results of the statistical analysis of interobserver agreement are shown in Table 1. The analysis showed moderate agreement among experts for the assessment of the recordings obtained before anaesthesia [*τ* = 0.47; 95% CI (0.21, 0.66)] and very high agreement for the assessment of the recordings obtained during anaesthesia [*τ* = 0.81; 95% CI (0.69, 0.89)] and 2 min after anaesthesia [*τ* = 0.89; 95% CI (0.82, 0.94)], as well as for the overall assessment of the recordings obtained before, during, and after anaesthesia [*τ* = 0.91; 95% CI (0.88, 0.94)] (36).

**Table 1:** Concordance coefficients for visibility ratings of individual ultrasound clips of the rat lung.

Recordings	Concordance coefficient^a^	*p*	95% *CI*	
			*LL*	*UL*
Overall	0.91	**<0.001**	0.88	0.94
Before	0.47	**0.025**	0.21	0.66
During	0.81	<0.001	0.69	0.89
After 2 min	0.89	**<0.001**	0.82	0.94

a Kendall’s tau concordance coefficient.

### Histopathological examination

**Figure 3:**
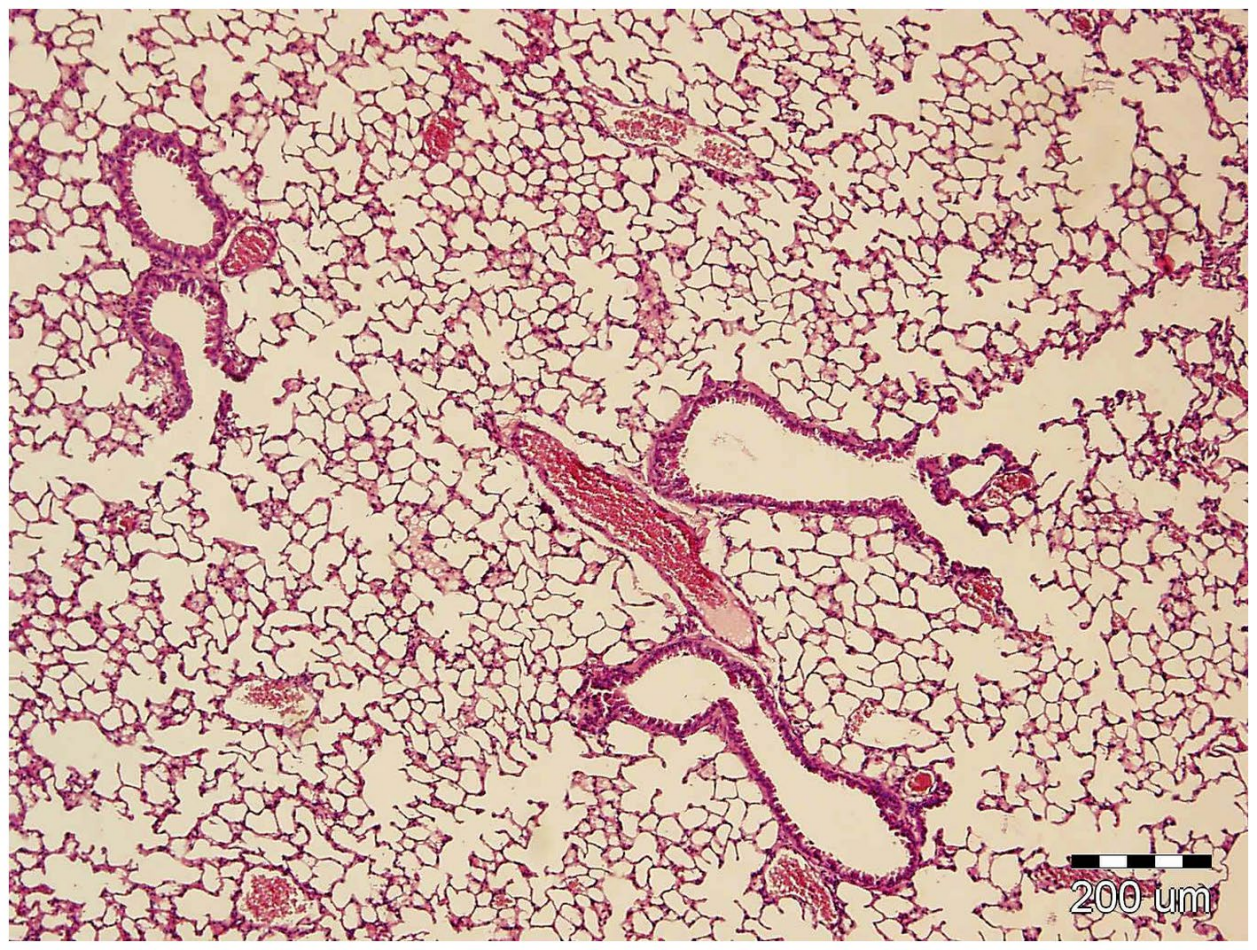
Hematoxylin and eosin (H&E) staining of the rat lung tissue. The lung tissue did not reveal any pathological alterations. The black scale bar represents 200 μm.

Histopathological examination of the lung tissue did not reveal any pathological alterations. Particular attention was paid to the presence of alveolar and interstitial changes, including any signs of alveolar infiltration or alveolar collapse, none of which were observed (Figure 3).

## Discussion

Lung ultrasound is a fast and non-invasive method that has been shown to be effective in detecting various intrathoracic pathologies in human and animal patients, including atelectasis (23, 24, 26, 27, 37–40). Anaesthesia-induced atelectasis can cause atelectrauma and a pro-inflammatory state, typically related to alveolar opening and collapse, potentially leading to damage of pulmonary capillaries and pneumocytes. This can lead to alveolar oedema, causing neutrophil activation and the release of inflammatory mediators (2, 41). In human medicine, atelectasis is usually not severe, but the collapsed lung can contain up to four times more tissue than aerated/ventilated lungs (42). In studies on postoperative pulmonary complications, atelectasis and pneumonia are usually mentioned together, as atelectatic changes may predispose to pneumonia (43). Another consequence of anaesthesia-induced atelectasis can be right heart failure, as described in a rat model (44). Surfactant function may also be impaired by volatile agents (isoflurane and sevoflurane) and cause atelectasis in rats (32). This could be a key factor in rapid atelectasis formation observed under isoflurane anaesthesia in the rats in this study.

Atelectasis is detected on ultrasound as consolidation, which can have various differential diagnoses (e.g., haemorrhage and pneumonia); therefore, ultrasound examination before anaesthesia is essential to differentiate anaesthesia-induced atelectasis from pre-anaesthetic pathology (45, 46). In the present study, the lungs were examined using the ultrasound protocol prior to induction of anaesthesia and were considered healthy (A-profile, aerated lungs). Another possible cause of atelectasis formation after inhalational anaesthesia induction is pulmonary capillary haemorrhage, which can lead to B-line formation, as described in rats and mice (47). The threshold for mechanical index (MI) should be set between 0.44 and 0.9 (48, 49). In the present study, a linear probe with an MI of 0.5–1.2 was used. Therefore, B-line formation can also result from capillary haemorrhage, but in studies of pulmonary capillary haemorrhage, exposure to the ultrasound wave takes much longer to generate B-lines. In addition, the ultrasound probe was positioned in the middle of the lung fields as described by Miller et al. (47) while gravity-dependent regions along the axillary lines were assessed in presented study. Pulmonary haemorrhage was then ruled out based on the histopathological examination results.

Anaesthesia-induced atelectasis can be detected at an early stage of anaesthesia (22, 50), which is consistent with the findings of the present study, where signs of early atelectasis formation were observed within the first 2 min of anaesthesia. In a study using thoracic CT in rabbits, all but one rabbit exhibited some degree of atelectasis, most likely related to positioning during the scan (51).

The modified version of Oricco’s LUSS was used in the present study (35). The modification of the LUSS was necessary because a single position of the probe allows scanning of the entire accessible lung surface within the imaging window. The Oricco scale was modified because a single probe placement in this species covers a relatively large area of the lungs. Consequently, at a single site, the difference between 25 and 50% involvement of the pleural line represents a substantial change, necessitating an additional subdivision for more precise scoring. Therefore, a point-based scoring system was not suitable, as the pathological process could affect a large part of the pleural surface. In human medicine, similar LUSS systems have been used in paediatric studies (52–54), suggesting the relevance of the modified version of Oricco’s LUSS for the assessment of anaesthesia-induced atelectasis. The analysis demonstrated strong interobserver agreement in the assessment of the recordings obtained during induction and 2 min after the onset of anaesthesia and moderate agreement among experts in the assessment of the recordings obtained before anaesthesia. In both lung areas, the operator’s evaluation showed that all animals had an LUSS of 0 (median 0, IQR 0–0) before anaesthesia. The moderate agreement in the pre-anaesthetic period might be due to the presence of a small number of vertical artefacts, which is considered a normal finding in conscious rats, but might be scored as 1 by another expert. These findings indicate that the modified LUSS and ultrasonographic assessment were highly reproducible and that diagnostic outcomes were consistent across independent examiners. The use of the modified LUSS in rats also showed that it can be used for the assessment of thoracic ultrasound in rats, providing a more precise definition of normal findings (e.g., A-lines with isolated vertical artefacts occupying less than 5% of the pleural surface).

A limitation of this study is the small number of animals used, as well as potential individual variability. Pulse oximetry monitoring was not performed, as this parameter can be affected by anaesthesia-induced atelectasis and performing instrument monitoring would have prolonged anaesthesia, which needed to be kept brief to perform lung ultrasound according to the study design.

In conclusion, anaesthesia-induced atelectasis can lead to severe changes detectable by lung ultrasound in gravity-dependent areas in a rat model anaesthetised with isoflurane in 95% oxygen delivered by an oxygen generator. Therefore, the use of isoflurane anaesthesia for respiratory imaging in this species should be approached with caution because of the potential involvement of gravity-dependent atelectasis (51). The modified version of Oricco’s LUSS showed significant interobserver agreement, indicating its potential reliability for thoracic ultrasound examinations in rats. As laboratory and pet rats are highly susceptible to respiratory infections and secondary *cor pulmonale* (55–57), lung ultrasound may not be ideal in rats anaesthetised with isoflurane in 95% oxygen via an anaesthetic mask, as anaesthesia-induced atelectasis can mimic serious pathologies and potentially be misinterpreted as pneumonic changes. Studies focusing on the safety of anaesthesia (including the anaesthesia protocol, (58–60)) and the prevention of atelectasis formation are very useful, as pre-existing pulmonary hypertension could be a complication leading to right heart failure during anaesthesia in both laboratory and pet rats.

## Data Availability

The original contributions presented in the study are included in the article/supplementary material, further inquiries can be directed to the corresponding author.

## Footnotes

1
www.random.org/sequences/
